# Co-Localization of DNA i-Motif-Forming Sequences and 5-Hydroxymethyl-cytosines in Human Embryonic Stem Cells

**DOI:** 10.3390/molecules24193619

**Published:** 2019-10-08

**Authors:** Yogini P. Bhavsar-Jog, Eric Van Dornshuld, Tracy A. Brooks, Gregory S. Tschumper, Randy M. Wadkins

**Affiliations:** 1 Department of Chemistry and Biochemistry, University of Mississippi, University, MS 38677, USA; bhavsar.yogini@gmail.com (Y.P.B.-J.); tschumpr@olemiss.edu (G.S.T.); 2 Department of Chemistry, Mississippi State University, Mississippi State, MS 39762, USA; edornshuld@chemistry.msstate.edu; 3 Department of Pharmaceutical Sciences, Binghamton University, Binghamton, NY 13902, USA; tbrooks@binghamton.edu

**Keywords:** DNA secondary structures, cytosine-rich DNA, DNA nanomaterials

## Abstract

G-quadruplexes (G4s) and i-motifs (iMs) are tetraplex DNA structures. Sequences capable of forming G4/iMs are abundant near the transcription start sites (TSS) of several genes. G4/iMs affect gene expression in vitro. Depending on the gene, the presence of G4/iMs can enhance or suppress expression, making it challenging to discern the underlying mechanism by which they operate. Factors affecting G4/iM structures can provide additional insight into their mechanism of regulation. One such factor is epigenetic modification. The 5-hydroxymethylated cytosines (5hmCs) are epigenetic modifications that occur abundantly in human embryonic stem cells (hESC). The 5hmCs, like G4/iMs, are known to participate in gene regulation and are also enriched near the TSS. We investigated genomic co-localization to assess the possibility that these two elements may play an interdependent role in regulating genes in hESC. Our results indicate that amongst 15,760 G4/iM-forming locations, only 15% have 5hmCs associated with them. A detailed analysis of G4/iM-forming locations enriched in 5hmC indicates that most of these locations are in genes that are associated with cell differentiation, proliferation, apoptosis and embryogenesis. The library generated from our analysis is an important resource for investigators exploring the interdependence of these DNA features in regulating expression of selected genes in hESC.

## 1. Introduction

DNA can adopt non-canonical conformations in its single-stranded (ssDNA) form, which can occur during the processes of replication, transcription and recombination. [1] Examples of such conformations are G-quadruplexes (G4s) formed from guanosine-rich ssDNA, and i-motifs (iMs) formed from cytosine-rich ssDNA [2,3]. The G4s are composed of arrays of planar guanosine quartets involved in Hoogsteen base-pairing, while iMs are composed of intercalated hemi-protonated cytosines [4,5]. The plasticity of DNA allows for these structures, which are usually referred to as secondary structures, to differentiate them from B-form DNA.

Genome-wide analyses of G4/iMs have revealed that these elements are concentrated proximal to the transcription start sites (TSS) of several genes and can potentially alter gene expression [6]. The occurrence of these structures in vivo remained a topic of great controversy for several decades [7]. However, the Balasubramaniam lab established the existence of G4s in living cells [8]. The iM structure was originally given much less consideration than the G4. While G4s can form at neutral pH, iMs require slightly acidic conditions (pH ~6.5), where the N1 position of cytosine can be protonated, allowing three hydrogen bonds to form between two cytosine residues in DNA [9]. The resulting four-stranded structure exhibits intercalated interactions between planes of cytosine base pairs, and therefore, has been referred to as iM DNA. In early studies, and in dilute solutions, at increasing pH, structural stability of iMs decreases to the point that at physiological pH (~7.3), little or no iM structure remains [9]. Hence, in the past, the iM has attracted less attention than G4s because the nucleus does not appear to be more acidic than the cytoplasm. However, the addition of crowding agents and/or dehydrating co-solvents can shift the pK_a_ for formation of an iM toward more physiological pH [10,11,12,13]. Longer C-rich sequences that form iMs at pH ~7 have been also reported [14]. Recently, iM structures have also been observed in cell nuclei, increasing interest in their possible biological role [15,16]. The factors affecting the dynamic behavior of G4s and iMs are being widely studied to understand the underlying mechanism of gene regulation by these DNA structures, but much remains to be unveiled.

In addition to the topological variation, the DNA in mammalian genomes undergoes epigenetic modification of cytosines to 5-methylcytosines (5mCs), 5-hydroxymethyl cytosines (5hmCs), and other higher oxidation states [17,18,19,20,21,22,23]. These epigenetic alterations are known to have implications in many biological processes, including DNA demethylation, transcription regulation, X-chromosome silencing, genomic imprinting, cell differentiation and tumorigenesis [24,25]. The DNA cytosines are enzymatically modified to 5-methylcytosines (5mC) by DNA methyl transferase, and can be further oxidized by ten eleven translocase (Tet) enzymes to yield 5hmC [26]. The 5-hydroxymethylated cytosines (often considered the sixth base in the mammalian genome) are prevalent in mammalian embryonic stem cells (ESC) [20,27]. Like G4/iMs, 5hmCs are also enriched near the TSS of several genes [20]. Intriguingly, analyses of the genome-wide distribution of 5hmCs in mouse ESC revealed that the presence of 5hmCs in promoters may preferentially contribute to gene repression, whereas intragenic 5hmCs contribute toward gene activation [27]. This suggests that, similarly to 5mC, 5hmC has a complex role to play in the process of gene regulation.

The fact that 5hmCs and iMs (*i*) are both enriched around the TSS of several genes and (*ii*) both function as gene regulatory elements led us to hypothesize that 5hmC and iMs may play an interdependent role in gene regulation. Hence, in the following study, we first evaluated the probability of proximal localization of iMs with 5hmCs in human ESC, and then identified a gene pool wherein 5hmCs are associated with the iM-forming sequences. We created a library of these genes that will be a resource for locating putative iM-forming sequences having 5hmCs associated with them in human ESC (Supplementary Materials). Accounting for the presence of epigenetic modifications on iMs may be crucial to understanding the role of these dynamic conformers in gene regulation. For example, the presence of 5hmCs and on iMs could not only alter the conformation and stability of these structures, but also alter their recognition by transcription factors and other biological molecules [28]. From our study, we conclude that very few iM-forming gene sequences have 5hmCs associated within putative iM structures. However, the genes that do have putative iM-forming sequences enriched in 5hmCs were found to be predominantly associated with cell differentiation, proliferation, apoptosis and embryogenesis-related processes. These data suggest that for selected genes, the two genetic phenomena may have interdependent roles in regulating gene expression in human ESC.

## 2. Results

### 2.1. Very Few iM-Forming Sequences Undergo 5-Hydroxymethylation

**Figure 1:**
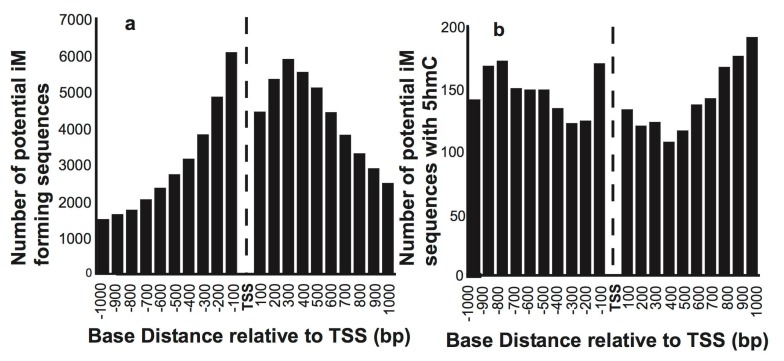
The overall distribution of the number of sequences that have putative iM-forming potential. (**a**) The number of sequences with putative iM potential and their position relative to the TSS of genes. (**b**) The number of putative iM-forming sequences that have 5hmCs co-localized within 100 bp of an iM relative to the TSS of genes.

We initially mapped the putative iMs in 15,760 reference sequence genes relative to the TSS. Figure 1a shows the number of genes having putative iM forming sequences in 100 bp segments relative to their TSS. As expected, most of the putative iM-forming sequences are concentrated in the proximity of the TSS of select genes. The histograms of G4/iM-forming sequences in human ESC that also have 5hmC localized within these putative G4s/iMs are plotted in Figure 1b, indicating that very few sequences associated with genes undergo 5hmC modifications in the vicinity of iMs/G4s. Moreover, the fraction of genes having 5hmC and iMs co-localized increases with the increasing distance from the TSS in both upstream and downstream directions.

### 2.2. 5hmCs and G4/iMs Distributions are Asymmetric around the TSS

**Figure 2:**
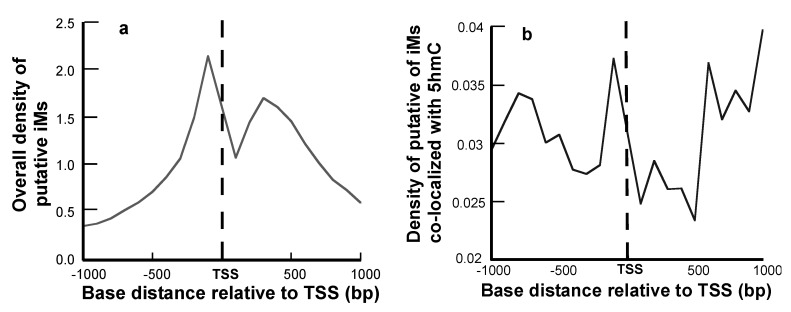
Positional dependence of iM and 5hmC localization. (**a**) The overall density of iM-forming sequences relative to the TSS. (**b**) The density of iM-forming sequences with 5hmCs co-localized within 100 bp of a putative iM.

**Figure 3:**
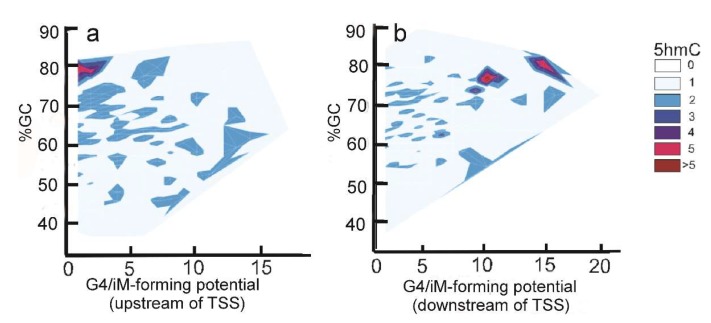
Relationship between iM potential (Equation (1) below) and 5hmC density. (**a**) The contour plot for the sequences upstream of the TSS shows that 5hmC enrichment is associated with sequences with low iM-forming potential. (**b**) The contour plot for the downstream sequences shows that the 5hmC enrichment is associated with sequences with high iM-forming potentials.

The density of iMs within 1 kb upstream and downstream was plotted for all the genes. The density-plot (Figure 2a) is in agreement with the prior work on plotting the frequency of iMs relative to the TSS [6,29]. Figure 2b shows the density of iMs for the genes with 5hmC co-localized near iMs. From these data, we infer that the genes undergoing 5hmC modification contribute very little to the overall genomic iM-density, and for the genes, the density curve shows a slight trend for 5hmC modification at locations distant from the TSS. Furthermore, the contour plots of 5hmC with respect to iM-forming potentials and GC content indicate that in the 1 kb region upstream relative to the TSS (Figure 3a), the 5hmC enrichment occurs around the sequences that have lower potential to form iMs. In contrast, in the 1 kb downstream region, the 5hmC enrichment occurs around the sequences with high iM-forming potential (Figure 3b). It should be noted that asymmetry in the distribution could be caused by intragenic regions that are more likely to be enriched in 5hmC content, such as CpG islands. The multivariate analyses to ascertain the overall correlation between iM-forming potential, number of 5hmC, and GC content in 1 kb upstream of the TSS is shown in Table 1, where iM-forming potentials and 5hmC content are weakly positively correlated to GC content. However, the 5hmC and iM potential shows a weakly negative correlation. For 1 kb downstream, the negative correlation between 5hmC and iMs disappears, and this region shows no statistically significant correlation between the two elements (Table 1).

### 2.3. Putative G4/iMs Enriched with 5hmC are Primarily Found in Differentiation and Proliferation Genes

**Figure 4:**
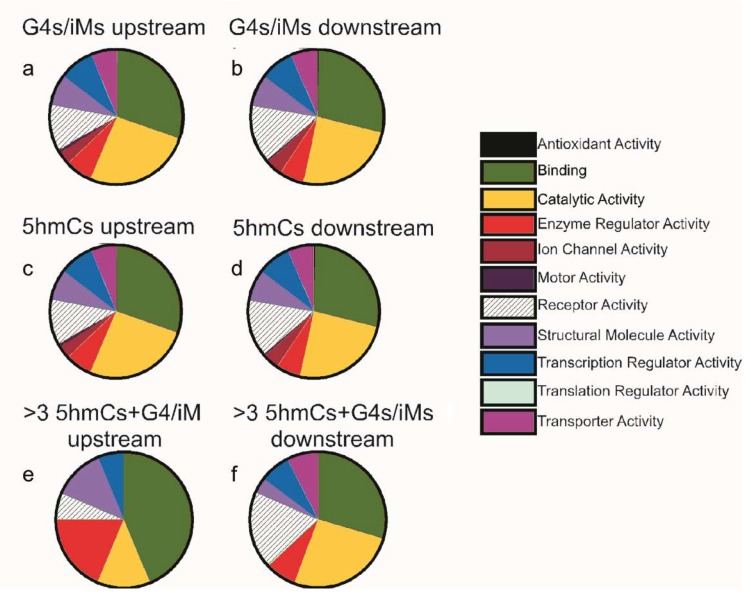
PANTHER pie charts showing the molecular function distributions of G4/iM-forming genes (irrespective of whether or not 5hmC-modifications) (**a**) upstream of TSS, and (**b**) downstream of TSS; 5hmC-containing genes (irrespective of presence of G4s/iM-forming sequences) (**c**) upstream and (**d**) downstream of the TSS; iM-forming genes that have 3 or more 5hmCs associated with them (**e**) upstream, and (**f**) downstream of the TSS.

From 15,760 genes, only 1222 sequences upstream and 1119 sequences downstream had 5hmC localized within 100 bp of G4/iM-forming sequences. Amongst these genes, there were 682 upstream (supplementary data S1) and 640 downstream (supplementary data S2) sequences that had 5hmC modification occurring on the cytosines involved in G4/iM-forming sequences. The 5hmC modification, as noted above, is not very common in sequences showing G4/iM-forming potentials. This led us to investigate whether the 5hmC modification is restricted to a class of genes involved in, or related to, specific molecular functions. Hence, the co-localized sequences were analyzed using the PANTHER classification tool, and the results are shown in Figure 4. The molecular function distributions of all the genes (irrespective of presence of 5hmC modification on them) having G4/iM-forming potentials were plotted for the upstream region from TSS (Figure 4a) and downstream relative to TSS (Figure 4b). Similarly, the function distributions of 5hmC-containing genes (irrespective of presence of G4s/iM-forming sequences) were plotted for both the upstream (Figure 4c) and downstream sequences (Figure 4d) relative to TSS. The pie charts for putative G4/iM-forming genes and 5hmC modified genes are very similar and indicate that these genes were mainly involved in binding and catalytic activities.

We further analyzed these sequences in order to evaluate the effects of increasing 5hmC-content on the molecular function of potential G4/iM-forming genes. Interestingly, this analysis revealed that sequences with three or more sites where 5hmC occurs within potential G4/iM-forming sequences upstream of the TSS genes (Table 2) are mainly involved in ligand binding (e.g., calcium, actin, and calmodulin binding) and enzyme regulatory activities (Figure 4e), and those downstream genes (Table 3) are associated with receptor activity (e.g., G-protein coupled and cytokine receptors; Figure 4f). Although these highly 5hmC-enriched genes in human ESC capable of forming iMs are associated with cell differentiation and proliferation or apoptosis, our analysis found that they are not confined to any single differentiation or apoptotic pathway.

We also performed functional enrichment analysis on these genes using DAVID to cluster the groups of genes associated with similar functional annotation terms [30]. The DAVID tool outputs clusters related to a particular biological process. Each cluster may be further composed of sub-groups of genes that show enrichment within the cluster. Our clustering results are shown in Table 4 (upstream of TSS) and Table 5 (downstream of TSS). For the genes located upstream of the TSS, “regulation” (enzyme regulation, metabolic activity regulation and negative regulation of transcription) is a significantly enriched term (*p* < 0.05). For the 5hmC enriched genes located downstream relative to the TSS, the “binding” term (ion binding and protein binding) is significantly enriched (*p* < 0.05). Further experiments may be warranted for these genes in order to evaluate the effects of 5hmC on G4/iM regulatory roles.

## 3. Discussion

DNA G4/iMs can enhance as well as suppress gene expression, making it a complex process to understand their exact mechanism of regulation. The G4/iMs are known to exist in several conformations, depending on the loop lengths and guanosine/cytosine stretches—a structural polymorphism that could explain the differential mechanism of gene regulation by them [31]. Amongst other factors, the presence of epigenetic modifications in the iMs and loop-regions of G4s could also influence the conformation and stabilities of these DNA topological variants, and hence might be one way by which both G4/iMs and 5hmCs could cooperate in the cellular environment. The presence of 5hmC on G4/iMs might modulate the binding abilities of transcription factors and other biomolecules by modifying the recognition sites on G4/iMs. For example, Sprujit et al. [21] demonstrated that different 5-modified cytosine derivatives (5mC, 5hmC, 5-formylcytosine, and 5-carboxycytosine) are recognized by distinctly different sets of transcription regulators and DNA-repair proteins in mouse ESC. In addition, we recently reported that 5hmC modification of the G4 from the VEGF promoter abrogates its recognition by nucleolin [28]. Hence, it may be imperative to account for the presence of 5hmC or other 5-substituted cytosines in loops of G4s and within iMs while studying gene regulation by these structures.

In this study, we compiled a library of human genes that have putative G4/iM-forming sequences epigenetically modified with 5hmC in ESC (Supplemental Materials) around their transcription start site (TSS). This library will readily facilitate the determination of the presence of 5hmCs in G4/iM-forming genes of interest. As an example of its use, from this library, we selected the G4/iM-forming that are 5hmC enriched (three or more 5hmC per G4/iM-forming sequence) to inspect the gene expression pathways in which they are involved. We found that CHRM1 (having putative G4/iMs in first 500 bp upstream), CYP27C1 and FOXH1 (having putative G4/iMs in their first 200 bp upstream) are all linked to pathways related to Alzheimer’s disease [32,33,34,35]. While our data pertains only to co-localization in ESC, our results may warrant investigation of the 5hmC modification and G4/iM formation in these genes in the progression of Alzheimer’s disease.

In the exercise of segregating and analyzing iM-forming sequences with three or more 5hmCs, we evaluated whether their related genes are involved in, or are related to, a particular class of molecular functions, biological processes, or pathways. This exercise serves as an example of how our library of putative iM-forming sequences with co-localized 5hmCs can be used with classification and clustering tools. It also constitutes a crucial part of our future work: since the data on genome-wide 5hmC modification on other cell types continues to emerge (e.g., [36]), it will prove interesting whether genes enriched in 5hmC in ESCs retain their 5hmC enrichment patterns in differentiated or cancer cells. In addition, under pathophysiological states, the pattern of 5hmC presence may be altered from that in ESC, and additional pathology-specific studies of G4/iM modification can be compared to the data in our library. The library will also be useful for comparison with 5hmC modification in G4/iM-forming regions in cancer stem cells [37].

Despite the fact that 5hmCs and G4/iMs are abundant near TSS of several genes, a very small fraction of sequences with G4/iM-forming potential showed the presence of 5hmC-modification. This led us to further evaluate the significance of this particular subset of genes for their physiological roles. We found that for those genes that have three or more 5hmC associated with the G4/iM-forming sequences and that are located upstream of the TSS, ligand binding activity and enzyme regulation were predominant molecular functions. For the genes with 5hmC enrichment located downstream from the TSS, ligand binding activity and receptor activities predominated. Closer inspection suggested that genes highly enriched in 5hmC are involved in cell differentiation, proliferation, apoptosis and embryogenesis. In future studies, it would be worthwhile to consider the presence of epigenetic modification of bases involved in G4/iM-forming sequences in order to comprehensively develop an understanding of possible interdependent regulatory roles of G4/iMs and 5hmC.

It should also be noted that we have previously reported that the presence of a single 5hmC in iM structures can significantly regulate the pH-dependent cooperativity of iM formation by C-rich DNA [10]. In the sequences reported in Table 2, as many as seven dC residues were found to be modified to 5hmC in certain sequences. It remains interesting to understand how 5hmC modification can affect not only the biological function of iMs, but also their use as a DNA nanomaterial.

## 4. Materials and Methods

Our technique to calculate iM density has been previously reported, and is summarized in [10]. To find the putative uni-molecular iMs, we implemented the Quadfinder tool developed by Scaria et al. [38]. This tool searches for sequences composed of C_x_N_y_C_x_N_y_C_x_N_y_C_x_ motifs (for iMs on template strands) or G_x_N_y_G_x_N_y_G_x_N_y_G_x_ motifs (for iMs on non-template strands), where x(=3–5) denotes the G/C stretch and y(=1–25) is the intervening loop length. The Quadfinder analyzes and lists all the probable motifs, including the overlapping ones, in a given DNA sequence. Promoters and intragenic regions of 15,760 reference sequence genes from the human GRCh37.p10 primary assembly were analyzed for the presence of iM/G4s. The promoter region is defined as a 1 kb stretch upstream of the TSS [29], while the intragenic analyses covered a 1 kb stretch downstream of the TSS. In order to account for the iMs present on template and non-template strands, the total numbers of iMs were calculated by summing the G-motifs and C-motifs found in the template strand. To calculate the density of iMs, the 1 kb regions upstream and downstream of TSS were divided into 100 bp segments for each gene, and each of these segments was analyzed with the Quadfinder. The density of iMs per gene in any 100 bp was then calculated using Equation (1):

(1) $$density\;of\;iM=\frac{\sum number\;of\;iM}{total\;number\;of\;genes\;analyzed}$$

The resulting plots are similar to those in prior published reports [6].

For localization of 5-hydroxymethylcytosines, we used the 5hmC sequencing data from H1 human ESC deposited to the Gene Expression Omnibus (accession GSE36173) by Yu et al. [23]. Their 5hmC sequencing was done using Tet-assisted bisulphite sequencing and was done on UCSC hg18 build. This data was converted by us to GRCh37 using the liftOver genome tool by UCSC [39]. The 5hmC density calculation is similar to the iM density calculation and is shown in Equation (2):

(2) $$density\;of\;5hmC=\frac{\sum number\;of\;5hmC}{total\;number\;of\;genes\;analyzed}$$

The correlation coefficients between G4/iM-potentials and GC content, 5hmC content and GC content, and G4/iM-forming potential and 5hmC content were calculated using JMP 10 statistical software. All three data columns (G4/iM-potentials, 5hmC and GC-content) were subjected to a multivariate analysis to compute the value for correlation coefficients; these coefficients were also estimated over the upper and the lower 95% confidence intervals. Since the correlation coefficients were very small, further analysis was done in JMP to assess whether or not these coefficients were significantly different from zero at the 0.05 significance level.

The molecular functions of the G4/iM-forming genes, the 5hmC-enriched genes, and the potential G4/iM-forming genes containing 5hmCs were assessed using the PANTHER (Protein ANalysis THrough Evolutionary Relationships) classification system, which is a part of the gene ontology reference genome project [40]. We did a manual search on each of the 5hmC-enriched iM-forming genes to evaluate their association with the terms “differentiation”, “proliferation”, “apoptosis”, “embryogenesis”, “transcription”, “translation”, “metabolism”, “biosynthesis”, “cytoskeletal”, “transport”, “ion binding”, and “enzyme” (Table 2 and Table 3). We also performed functional enrichment analysis on these genes using the Database for Annotation, Visualization, and Integrated Discovery (DAVID) bioinformatics resources to cluster the groups of genes associated with similar functional annotation terms [30]. The DAVID tool measures relationship between the annotation terms based on the degrees of their co-association in order to group the similar, redundant and heterogeneous annotation contents into annotation clusters. Only the groups of genes that showed statistically significant enrichment in a particular functional annotation (*p*-value < 0.05) are listed in Table 4 and Table 5.

## Figures and Tables

**Table 1:** Correlations between number of 5hmC, iM-forming potential, and GC-content in 1 kb region upstream and downstream of TSS. The correlations were also computed for the upper and lower 95% confidence interval range. Since the correlation coefficients are very small, further analysis was done in order to assess whether or not these coefficients were significantly different from zero at a significance level of 0.05. The (*) indicates that the correlation significantly differs from being zero.

1 kb upstream of TSS		Correlation	Lower 95%	Upper 95%	Signif. Prob
iM Potential	GC content	0.29	0.25	0.34	<0.0001 *
5hmC content	GC content	0.12	0.07	0.17	<0.0001 *
5hmC content	iM Potential	−0.07	−0.12	−0.02	0.01 *
**1 kb downstream of TSS**					
iM Potential	GC content	0.30	0.25	0.34	<0.0001 *
5hmC content	GC content	0.06	0.01	0.10	<0.0293 *
5hmC content	iM Potential	−0.02	−0.07	0.03	0.4782

**Table 2:** Functional classification of iM-forming genes with three or more 5hmCs within a G4/iM-forming sequence upstream of the TSS. The number of 5hmCs present is given in parenthesis.

Differentiation Proliferation Apoptosis	Embryogenesis	Transcription Translation	Metabolism/Biosynthesis Cytoskeletal Organization Transport/Ion Binding Enzyme Activity
**BIRC7 (5)** protein binding, peptidase inhibitor activity	**CST3 (4)** protein binding	**MRPS24 (3)** structural constituent of ribosome, nucleic acid binding	**BAIAP2L2 (5)** receptor activity
**CHRM1 (4)** G-protein coupled receptor activity	**DTX1 (3)** developmental process	**C17orf49 (3)** nucleic acid binding	**CDIPT (3)** transferase activity
**CST3 (4)** protein binding, cysteine-type endopeptidase inhibitor activity	**DUSP2 (3)** phosphoprotein phosphatase activity, protein binding, kinase inhibitor activity, kinase regulator activity	**SORBS3 (3)** structural constituent of cytoskeleton	**CYP27C1 (3)** oxidoreductase activity
**CYGB (4)** blood circulation, transport	**FOXH1 (5)** transcription factor activity	**ZGPAT (3)** Negative regulation of transcription	**EPS8L2 (3)** intracellular signaling cascade, cell motion intracellular signaling cascade
**DTX1 (3)** developmental process	**PLEC (4)** structural constituent of cytoskeleton, calcium ion binding actin binding	**FOXH1 (5)** transcription factor activity	**ITIH4 (3)** protein binding, serine-type endopeptidase inhibitor activity
**DUSP2 (3)** phosphoprotein phosphatase activity, protein binding, kinase inhibitor activity, kinase regulator activity	**TNFSF13 (3)** cytokine binding, Tumor necrosis factor receptor binding	**MAMSTR (4)** transcription factor binding, nucleic acid binding	**KCTD6 (3)** protein binding
**FOXH1 (5)** transcription factor activity	**PRR15L (3)** Negative regulation of transcription		**MPDU1 (3)** lipid metabolic process, protein amino acid glycosylation
**HSPBP1 (4)** protein binding, enzyme regulator activity			**PADI3 (3)** hydrolase activity
**MAMSTR (4)** transcription factor binding nucleic acid binding			
**NHP2 (4)** structural constituent of ribosome, nucleic acid binding			
**PRR15L (3)** Negative regulation of transcription			
**RASGRP4 (4)** calcium ion binding, receptor binding, small GTPase regulator activity, guanyl-nucleotide exchange factor activity			

**Table 3:** Functional classification of iM-forming genes with three or more 5hmCs within a G4/iM-forming sequence downstream of the TSS. The number of 5hmCs present is given in parenthesis.

Differentiation Proliferation Apoptosis	Embryogenesis	Transcription Translation	Metabolism/Biosynthesis Cytoskeletal Organization Transport/Ion Binding Enzyme Activity
**CTCFL (7)** transcription factor activity	**H1FOO (3)** DNA binding	**VAV1(3)** receptor binding, small GTPase regulator activity, guanyl-nucleotide exchange factor activity	**EMILIN1(3)** cell adhesion
**ENG (4)** transforming growth factor, beta receptor activity, cytokine receptor activity	**HHIPL1(3)** G-protein coupled receptor activity		**EPS8L1 (4)** intracellular signalling cascade cell motion, intracellular signalling cascade
**GPR55(3)** G-protein coupled receptor activity	**SCUBE2** visual perception, sensory perception, signal transduction, cell-cell adhesion, mesoderm development		**IP6K3 (6)** kinase activity
**H1FOO (3)** DNA binding	**VIL1(3)** structural constituent of cytoskeleton, actin binding		**MFSD4(3)** transport
**HHIPL1(3)** G-protein coupled receptor activity			**NME4 (4)** nucleotide kinase activity
**PLCB2(3)** phospholipase activity, calcium ion binding, receptor binding, small GTPase regulator activity, guanyl-nucleotide exchange factor activity			**S100A16 (3)** calcium ion binding, receptor binding calmodulin binding
**SCUBE2(3)** visual perception, sensory perception, signal transduction, cell-cell adhesion, mesoderm development			**SLC25A23 (3)** amino acid transmembrane, transporter activity, transmembrane transporter activity, calcium ion binding, calmodulin binding
**SP100 (3)** transcription factor activity chromatin binding, receptor binding, transcription factor activity			**SLC37A1 (3)** cation transmembrane, transporter activity
**VIL1(3)** structural constituent of cytoskeleton, actin binding			**TYROBP (3)** receptor activity
**VAV1(3)** receptor binding, small GTPase regulator activity, guanyl-nucleotide exchange factor activity			**DPEP1** Metalo-peptidase activity

**Table 4:** DAVID clustering of biological processes of iM-forming genes with three or more 5hmCs within a G4/iM-forming sequence upstream relative to TSS. The statistical significance of these processes being enriched versus random gene selection is indicated by the *p* value shown.

Biological Processes Cluster 1			Biological Processes Cluster 2	
Regulation of Protein Kinase Activity (*p* = 0.02)	Regulation of Kinase Activity (*p* = 0.02)	Regulation of Transferase Activity (*p* = 0.02)	Negative Regulation of Macromolecular Metabolic Processes (*p* = 0.03)	Negative Regulation of Transcription (*p* = 0.04)
ZGPAT	ZGPAT	ZGPAT	PRR15L	PRR15L
BIRC7	BIRC7	BIRC7	CST3	FOXH1
CHRM1	CHRM1	CHRM1	FOXH1	SORBS3
DUSP2	DUSP2	DUSP2	SORBS3	ZGPAT
			ZGPAT	

**Table 5:** DAVID clustering of biological processes of iM-forming genes with three or more 5hmCs within a G4/iM-forming sequence downstream relative to TSS. The statistical significance of these processes being enriched versus random gene selection is indicated by the *p* value shown.

Biological Processes Cluster 1		Biological Processes Cluster 2	
Metal Ion Binding (*p* = 0.01)	Calcium Ion Binding (*p* = 0.004)	Identical Protein Binding (*p* = 0.04)	Protein Homodimerization Activity (*p* = 0.06)
CTCFL	S100A16	S100A16	S100A16
S100A16	PLCB2	SP100	SP100
SP100	SCUBE2	EMILIN1	ENG
NME4	SLC25A23	ENG	
PLCB2	VIL1		
SCUBE2			
SLC25A3			
VAV1			
VIL1			
DPEP1			

## Supplementary Materials

The following spreadsheets are available online, S1-upstream-5hmC.xls, and S2-downstream-5hmC.xls
